# Induction of Neuronal Death by Microglial AGE-Albumin: Implications for Alzheimer’s Disease

**DOI:** 10.1371/journal.pone.0037917

**Published:** 2012-05-25

**Authors:** Kyunghee Byun, Enkhjaigal Bayarsaikhan, Daesik Kim, Chae Young Kim, Inhee Mook-Jung, Sun Ha Paek, Seung U. Kim, Tadashi Yamamoto, Moo-Ho Won, Byoung-Joon Song, Young Mok Park, Bonghee Lee

**Affiliations:** 1 Center for Genomics and Proteomics, Lee Gil Ya Cancer and Diabetes Institute, Gachon University of Medicine and Science, Incheon, Korea; 2 Department of Anatomy and Cell Biology, Gachon University of Medicine and Science, Incheon, Korea; 3 Department of Biochemistry & Biomedical Sciences, Seoul National University College of Medicine, Seoul, Korea; 4 Department of Neurosurgery, Seoul National University College of Medicine, Seoul, Korea; 5 Department of Medicine, University of British Columbia, Vancouver, Canada; 6 Structural Pathology, Institute of Nephrology, Graduate School of Medical and Dental Sciences, Niigata University, Niigata, Japan; 7 Department of Anatomy and Neurobiology, and Institute of Neurodegeneration and Neuroregeneration, College of Medicine, Kangwon National University, Chuncheon, Korea; 8 Laboratory of Membrane Biochemistry and Biophysics, National Institute on Alcohol Abuse and Alcoholism, National Institutes of Health, Bethesda, Maryland, United States of America; 9 Mass Spectrometer Research Center, Korea Basic Science Institute, Chungcheong-bukdo, Korea; Thomas Jefferson University, United States of America

## Abstract

Advanced glycation end products (AGEs) have long been considered as potent molecules promoting neuronal cell death and contributing to neurodegenerative disorders such as Alzheimer’s disease (AD). In this study, we demonstrate that AGE-albumin, the most abundant AGE product in human AD brains, is synthesized in activated microglial cells and secreted into the extracellular space. The rate of AGE-albumin synthesis in human microglial cells is markedly increased by amyloid-β exposure and oxidative stress. Exogenous AGE-albumin upregulates the receptor protein for AGE (RAGE) and augments calcium influx, leading to apoptosis of human primary neurons. In animal experiments, soluble RAGE (sRAGE), pyridoxamine or ALT-711 prevented Aβ-induced neuronal death in rat brains. Collectively, these results provide evidence for a new mechanism by which microglial cells promote death of neuronal cells through synthesis and secretion of AGE-albumin, thereby likely contributing to neurodegenerative diseases such as AD.

## Introduction

Alzheimer’s disease (AD) is the one of the most prevalent neurodegenerative diseases in humans. After the early observations on the activated microglia and its relation to AD [1]–[3], numerous reports indicated that chronic inflammatory processes contribute to the pathology of AD [4], [5]. One of the main central hypotheses is that the activated microglial cells cause neuronal damage and contribute to neurodegenerative changes in AD.

After a pilot study of Human Brain Proteome Project, we recently reported that albumin can be synthesized in microglial cells in the brain. We also demonstrated that the synthesis and extracellular secretion of albumin from microglial cells is enhanced upon microglial activation following Aβ1–42 exposure [6]. We initially proposed that albumin production would be beneficial to the cells by suppressing Aβ polymerization with enhancement of its clearance [6]. However, the precise role of albumin synthesized in the brain is still unknown.

Glycation reaction represents a post-translational modification process between free reducing sugars and free amino groups in many proteins. Advanced glycation end-products (AGEs), irreversible adducts of the Maillard reaction, have been demonstrated to accumulate in the brain during the course of ageing [7]. In fact, several reports showed increased AGE levels in the brains of AD individuals, suggesting pathological roles of AGEs in neurodegenerative disorders including AD, where markedly activated microglial cells and Aβ deposition colocalized with AGEs [8]–[24]. However, despite these reports, the pathological role and detailed mechanism of AGEs in promoting neuronal cell death and neurodegeneration are poorly understood. We hypothesized that secreted AGEs promote death of neuronal cells through activating the stress-activated protein kinases, which further activate cell-death associated Bcl-2 homolog proteins, in the primary neuronal cells and the brains from AD individuals. The aims of this study were to determine whether human primary microglial cells synthesize AGEs as AGE-albumin and to investigate the mechanism by which secreted AGE-albumin promotes death of primary neuronal cells, rat brains treated with Aβ1–42 peptide and human brains from AD individuals. Our results demonstrate that AGE-albumin is not only synthesized in microglial cells but also promotes death of neuronal cells in primary culture, Aβ1–42-exposed rat brains, and the brains of AD individuals, ultimately contributing to neurodegeneration.

## Results

### 1. Ubiquitous Distribution of AGE-albumin in the Brains of Human AD Individuals and Aβ-exposed Rats

**Figure 1:**
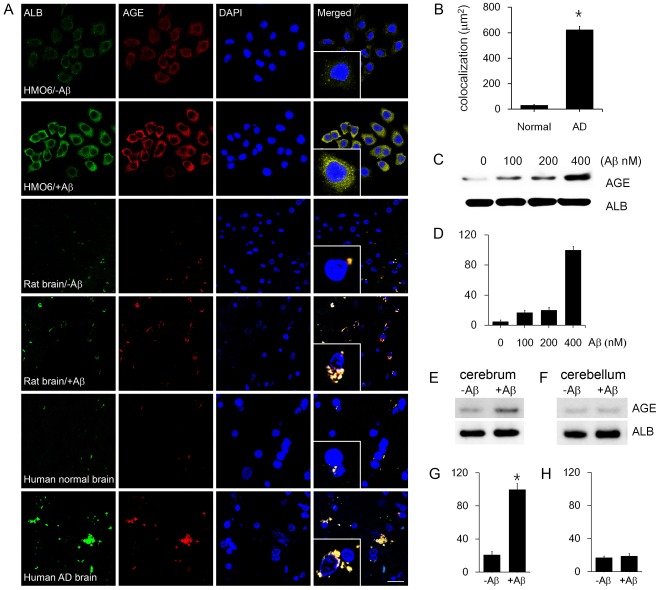
Distribution and synthesis of AGE-albumin in microglial cells and rat or human brains. (A) Triple-labeled confocal microscopic image analyses were used to study the distribution and relative levels of albumin (green), AGE (red) and DAPI (blue) in the HMO6 microglial cells and entorhinal cortex of rat brains before or after Aβ treatment as well as cerebral cortex of human brains from normal or AD individuals. HMO6 cells and rats were treated with Aβ1–42 described in the Materials and Methods section. Scale bar = 50 µm. These results represent similar images of 5 independent analyses. (B) The AGE-albumin positive particles in cortex of human AD brain were significantly different from the normal brains (p<0.05), as determined by densitometric analysis using Zeiss Zen 2009 software. (C, D) The degree of AGE-albumin synthesis in HMO6 cells was determined by immunoblot analysis (C) and densitometric analysis (D) after exposure to different concentrations of Aβ1–42, as indicated. The level of albumin is shown as an internal control for equal protein loading per lane. (E-H) The immunoblots of AGE-albumin in rat cerebrum (E, G) and cerebellum (F, H), with or without Aβ1–42 treatment and densitometric analyses, are shown. *, significantly different (*P*<0.001) from the level of AGE-albumin without Aβ1–42 treatment.

To study the mechanisms by which AGE-albumin synthesis is increased while how it promotes neuronal cell death, we first investigated the distribution of AGE and albumin in HMO6 microglial cells. Surprisingly, most AGEs were co-localized with albumin, suggesting that AGE-albumin could be a major AGE product in microglial cells of the brain (Fig. 1A). To further demonstrate the co-localization of AGE with albumin, we performed double immunohistochemical staining in human HMO6 microglial cells before and after Aβ treatment. AGE levels were markedly increased after Aβ exposure and most AGEs were co-localized with albumin. In addition, the tissue levels of AGE and albumin were strikingly elevated, and AGE was co-localized with albumin in Aβ–treated rat brains and human brains of AD individuals compared with control rat and human brains, respectively (Fig. 1A). Densitometric analysis indicated a 17.9-fold increase in AGE-albumin in the brains of individuals with AD (n = 5) compared to samples from normal individuals (n = 5) (Fig. 1B). Interestingly, the double-labeled AGE-albumin immunoreactive material was highly localized in the vicinity of cells with apoptotic nuclei. This strongly indicates that AGE-albumin may be directly involved in cell death in the brain. Immunoblot analysis of whole cell lysates revealed that the rate of AGE-albumin synthesis in HMO6 microglial cells was markedly and concentration-dependently increased following Aβ exposure (Figs. 1C, D). Moreover, immunoblot analysis of rat brain before and after Aβ treatment revealed that the amount of AGE-albumin increased significantly in cerebrum but not in cerebellum after Aβ treatment into the rat entorhinal cortex (Figs. 1E–H).

### 2. Synthesis and Secretion of AGE-albumin in Microglial Cells

**Figure 2:**
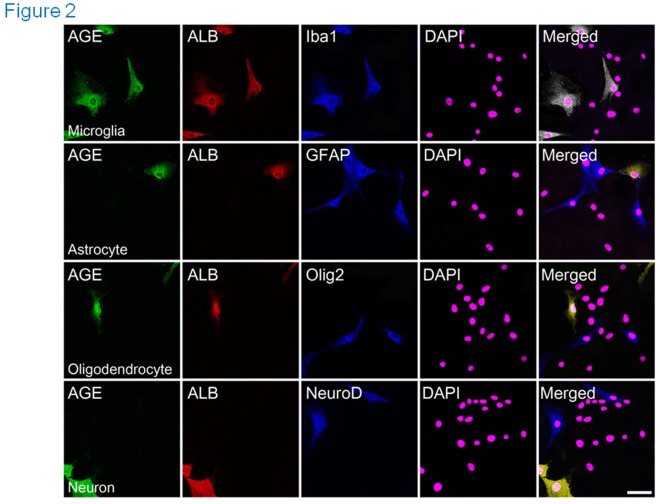
Synthesis of AGE-albumin in microglial cells but not from astrocytes, oligodendrocytes or neurons from human primary brain cells. (A) Triple-labeled fluorescent microscopic image analyses were used to demonstrate co-localization of AGE (green), albumin (red), and a specific marker of different cells (blue) in human primary brain cells. Representative images of microglial cells (Iba1), GFAP (an astrocyte marker), Olig2 (an oligodendrocyte marker), and NeuroD (a neuronal marker) in the human primary brain cells are shown. Similar results were observed in 5 independent analyses. Scale bar = 50 µm.

Because of the elevated levels of AGE-albumin in 3 different experimental models, we further investigated the cell-specific distribution of AGE-albumin in the human primary brain cells. The microglial marker, Iba-1, was generally co-expressed with AGE and albumin. In contrast, only limited amounts of the astrocyte marker (GFAP), the oligodendrocyte marker (Olig2), and the neuronal marker (NeuroD) were co-localized with AGE-albumin in human primary brain cells (Fig. 2). Based on these results, we concluded that AGE-albumin, the most abundant protein modified by AGE, is produced largely by microglial cells of the human brain.

**Figure 3:**
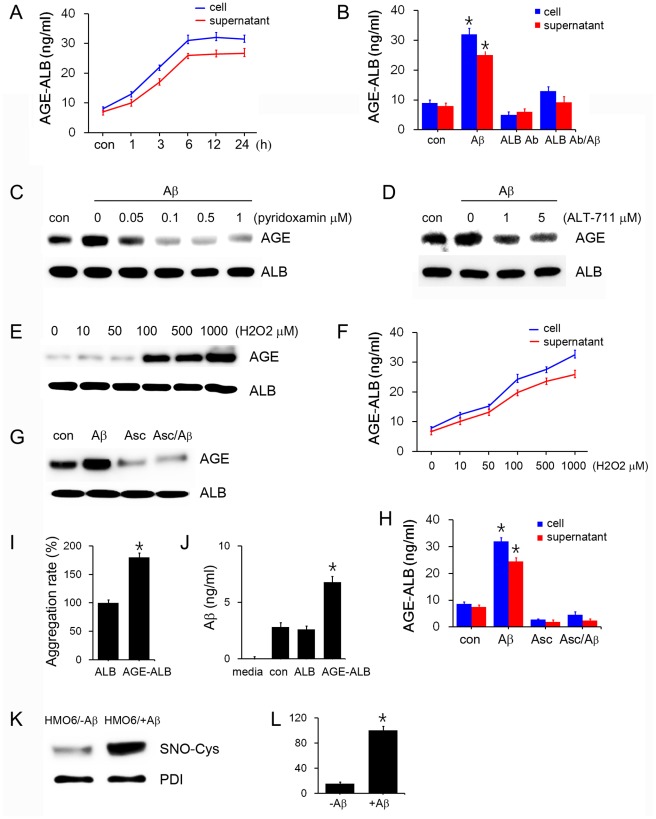
Synthesis of AGE-albumin in human microglial cells and its extracellular secretion. (A) The time-dependent changes in intracellular (cell lysate) and extracellular (supernatant) AGE-albumin in HMO6 cells, treated with Aβ1–42 for 1, 3, 6, 12, 24 h, were determined by ELISA. (B) The amounts of intracellular (cell lysate) and extracellular (supernatant) AGE-albumin in HMO6 cells, exposed to 3 different conditions as indicated, were determined by ELISA. The microglial cells were treated with: Aβ1–42 alone (5 nM) for 6 h, anti-albumin antibody (ALB Ab, 1 µM) for 24 h, or Aβ1–42 treatment after exposure to anti-albumin antibody overnight. (C-H) The amounts of AGE-albumin were determined by immunoblot analysis of the whole cell lysates of HMO6 cells exposed to different concentrations of pyridoxamine (from 0 to 1 µM) (C), ALT-711 (from 0 to 5 µM) (D), hydrogen peroxide (from 0 to 1,000 µM) (E, F) or 5 µM ascorbic acid (G, H) for 6 h in the absence or presence of 5 nM Aβ1–42 peptide. (I) Relationship between AGE-albumin and Aβ, contributing to Aβ aggregation. Aβ aggregation rates in HMO6 cells, treated with albumin alone or AGE-albumin, were determined by ThT fluorescence analysis. (H) HMO6 cells were exposed to albumin (ALB) or AGE-albumin (AGE-ALB) for 24 h. The respective amounts of Aβ in the culture media from untreated and AGE-albumin-treated cells were measured by ELISA. (I, J) Increased *S*-nitrosylation of PDI in HMO6 cells after treatment with Aβ. Microglial cells were exposed to Aβ1–42 (400 nM) for 6 h. PDI in whole cell lysates (0.4 mg protein/sample) was immunoprecipitated with the specific antibody. The immunoprecipitated PDI was subjected to immunoblot analysis with anti-*S*-NO-Cys or anti-PDI antibody. *, Significantly different from control or albumin-treated cells by densitometric analysis (p<0.05).

Next, we evaluated whether AGE-albumin secretion was increased when human HMO6 microglial cells were activated with Aβ. Immunoblot analysis of whole cell lysates and ELISA data for the culture media showed that AGE-albumin synthesis in human microglial cells and its extracellular secretion were significantly elevated by 1.6 times after Aβ treatment time-dependently (Figs. 3A, B). When cells were treated with the specific antibody against albumin, the amount of AGE-albumin was decreased regardless of Aβ treatment.

### 3. Increased Synthesis and Secretion of AGE-albumin by Oxidative Stress in Human Microglial Cells

We investigated whether the AGE-albumin synthesis and secretion are directly reduced by AGE inhibitor (pyridoxamine) or AGE cross-link breaker (ALT-711). When the HMO6 microglial cells were exposed to pyridoxamine or ALT-711, the amount of AGE-albumin was dramatically reduced in a concentration-dependent manner (Figs. 3C, D).

Next, we investigated whether the AGE-albumin synthesis and secretion are directly induced by elevated oxidative stress. When the HMO6 microglial cells were exposed to a strong oxidant, hydrogen peroxide, the amount of AGE-albumin was increased in a concentration-dependent manner (Figs. 3E, F). In contrast, addition of an anti-oxidant ascorbic acid (Asc) dramatically decreased the amount of AGE-albumin regardless of Aβ treatment (Figs. 3G, H). These data suggest that the amounts of both intracellular and secreted AGE-albumin, but not albumin itself, positively correlated with the degree of oxidative stress. Based on these findings, we conclude that the Aβ–induced synthesis of AGE-albumin in human HMO6 microglial cells and its extracellular secretion are closely related to increased oxidative stress.

### 4. Aβ Polymerization and Increased Aβ Synthesis by AGE-albumin in Human Microglial Cells

To determine whether AGE-albumin increases Aβ synthesis and accumulation in human microglial cells compared to albumin, we determined the amounts of Aβ, beta-amyloid cleavage enzyme (BACE), ADAM10 and APP by immunohistochemistry, immunoblot analysis, and ELISA respectively. BACE levels were markedly increased in AGE-albumin-exposed HMO6 cells compared to untreated or albumin-treated cells whereas the levels of ADAM10 or APP were unchanged (Figure S1).

We next studied the functional role of albumin or AGE-albumin in Aβ aggregation by staining with thioflavin T (ThT), which reflects the degree of Aβ aggregation. The ThT fluorescence assay revealed that the aggregation of Aβ was significantly increased by 1.6 times after addition of AGE-albumin than that with albumin alone (used as 100% control) (Fig. 3I). ELISA analysis revealed that the amount of Aβ in culture media was dramatically increased after HMO6 cells were exposed to AGE-albumin compared to untreated cells or albumin-treated cells (Fig. 3J).

We also hypothesized that increased Aβ accumulation may be promoted by inactivation of a chaperone PDI (protein disulfide isomerase), which has been shown to be inactivated by *S*-nitrosylation in the brains of AD individuals, leading to Aβ accumulation [17]. Immunoblot results of the immunoprecipitated PDI (55 kDa) revealed that the level of *S*-nitrosylated PDI was increased in Aβ-exposed HMO6 cells compared to that in untreated control (Figs. 3K, L). These results suggest that AGE-albumin increases microglial Aβ synthesis and accumulation in a vicious cycle, which further aggravates the AD conditions through increased Aβ production via up-regulating the BACE level, AGE-albumin synthesis, and neuronal cell death.

### 5. Induction of RAGE (Receptor of Advanced Glycation End Product) and Promotion of Neuronal Apoptosis by AGE-albumin

**Figure 4:**
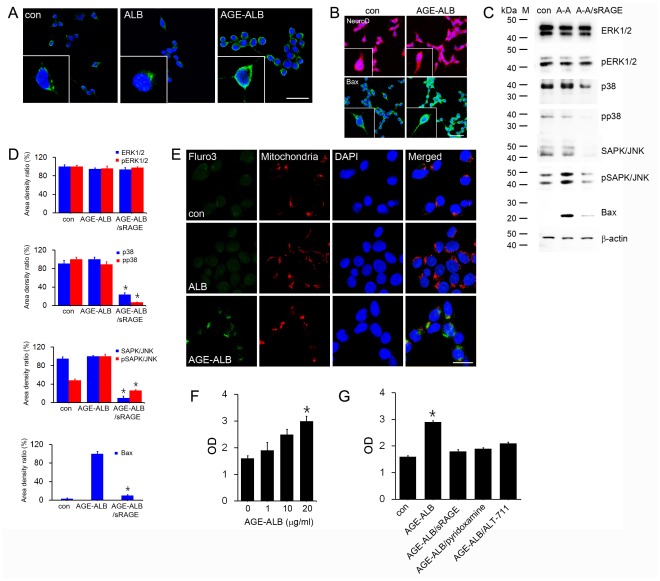
Induction of neuronal cell death by AGE-albumin through up-regulation of RAGE, mitochondrial calcium influx, and MAPK-Bax pathway. (A) The relative levels of RAGE (green) or DAPI (blue) in human primary neuronal cells, before or after albumin (ALB) or AGE-albumin (AGE-ALB) treatment for 6 h, were evaluated by double confocal microscopic image analyses. Similar results were observed in 5 independent analyses. (B) Double confocal microscopic images simultaneously show the neuronal marker (NeuroD) and relative levels of Bax (green), or DAPI (blue) in human neuronal cells before or after AGE-ALB treatment for 6 h. (C, D) Whole cell lysates (0.01 mg protein/lane) of human neuronal cells, before or after AGE-albumin exposure, were subjected to immunoblot analysis to determine the levels of ERK1/2, pERK1/2, p38, pp38, pSAPK/JNK, and Bax with specific molecular weight markers (M). β-Actin was used as an internal control for equal protein loading for each lane. (E) Increased level of mitochondrial calcium was evaluated by triple labeled confocal microscopic image analysis before (top panel) and after human neuronal cells were exposed to ALB (middle) or AGE-albumin (bottom): calcium concentration (Fluor-3, green), mitochondria (red), or DAPI-stained nuclei (blue). Scale bar = 50 µm. These results represent similar images of 5 independent analyses. (F, G) The rate of cell death was determined by the apoptosis assay after human neuronal cells were exposed to different concentrations of AGE-albumin alone. (F), or 20 µg/mL AGE-albumin treatment in the absence or presence of co-treatment with sRAGE, pyridoxamine or ALT-711 for 24 h (G).

A receptor protein for AGEs (RAGE) is known to be expressed in neurons, while its increased level is highly correlated with neuronal death and development of AD [16]. In addition, AGE binds to RAGE in primary neurons [25]. Therefore, we also assessed whether AGE-albumin can increase production of RAGE, a strong indicator of neuronal apoptosis in AD [26], [27], leading to cell death in human primary neurons. Immunohistochemical and immunoblot data showed that the amount of RAGE was significantly increased in primary neurons exposed to AGE-albumin compared to those untreated or treated with albumin only (Fig. 4A).

Since the stress-activated MAPKs and increased mitochondrial calcium influx are critically important in initiating apoptosis [18], [19], we monitored the changes in the respective levels of MAPKs, Bax, mitochondrial calcium influx, and cell death rate in the human primary neurons. Immunoblot analyses showed that the levels of pSAPK/JNK, and Bax were significantly increased after the human neuronal cells were exposed to AGE-albumin compared to control (albumin alone). But p38K, pp38K, SAPK/JNK, pSAPK/JNK and Bax levels were decreased dramatically after the human neuronal cells were exposed to AGE-albumin and sRAGE (Figs. 4B–D). Microscopic images showed the same pattern of elevated levels of Bax, which are co-localized with NeuN and DAPI-stained apoptotic neuronal cells in human AD brains compared with normal brain tissues (data not shown). In addition, mitochondrial imaging analysis of living cells showed that mitochondrial calcium concentration in human neuronal cells was increased in a time-dependent manner following exposure to AGE-albumin (Fig. 4E).

Consistent with these results, the optical density for apoptotic neuronal cells by apoptotic assay increased gradually in a dose-dependent manner after AGE-albumin exposure (µg/mL range) (Fig. 4F). In contrast, very few neuronal cells died in the presence of albumin alone (mg/mL range) (data not shown). Furthermore, the apoptosis assay showed that apoptosis of neuronal cells increased after AGE-albumin treatment, but remains same after co-treatment with AGE-albumin and sRAGE, pyridoxamine or ALT-711 (Fig. 4G). These data demonstrate that AGE-albumin directly promotes apoptosis of neuronal cells through activating the calcium-JNK-Bax pathway, as demonstrated previously in different cell types [28], [29].

### 6. Protection by Soluble RAGE (sRAGE) Against Aβ-mediated Neuronal Death

**Figure 5:**
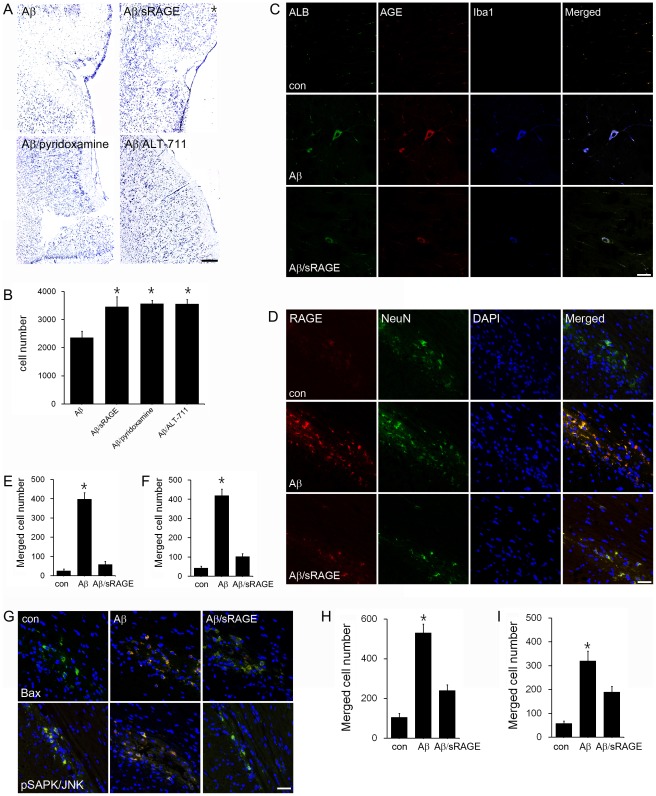
Protection of Aβ-mediated neuronal cell death by sRAGE, pyridoxamine or ALT-711 through decreasing RAGE levels. (A, B) The relative levels of neurons in rat brains were evaluated by cresyl violet staining after Aβ injection without or with sRAGE, pyridoxamine or ALT-711 co-treatment for 72 h. *, Significantly increased in Aβ/sRAGE, Aβ/pyridoxamine and Aβ/ALT-711 co-treated samples compared to Aβ treatment only (p<0.01). (C) Triple confocal microscopic images simultaneously show the relative numbers of AGE, albumin, or Iba1 positive cells in the rat entorhinal cortex (EC) before or after Aβ or Aβ/sRAGE injection for 72 h. These results represent similar images of 5 independent analyses. (D) *, The number of the triple-labeled cells (AGE/albumin/Iba-1 positive cells) significantly increased in whole EC area of Aβ injected rat brain but decreased dramatically in Aβ/sRAGE treated rat brain (p<0.01). (E) The levels of RAGE positive neuronal cells were evaluated by triple-labeled confocal microscopic image analysis in entorhinal cortex of control, Aβ, or Aβ/sRAGE injected rat brains. (F) *, The number of RAGE positive neuronal cells significantly increased in Aβ injected rat brain but decreased dramatically in Aβ/sRAGE treated rat brain (p<0.01). (G-I) The number of Bax or pSAPK/JNK positive neurons was evaluated by confocal microscopy. (H, I) *, The number of Bax positive neuronal cells (H) or pSAPK/JNK positive neuronal cells (I) significantly increased in Aβ injected rat brain but decreased dramatically in Aβ/sRAGE treated rat brain (p<0.01). Scale bar = 50 µm.

To investigate the protective effect of sRAGE against Aβ-mediated neuronal death, we performed *in vivo* analysis after injection of Aβ alone or co-injection of Aβ and sRAGE (Aβ/sRAGE), pyridoxamine (Aβ/pyridoxamine) or ALT-711 (Aβ/ALT-711) into rat brain. The relative levels of neurons in rat brains were dramatically increased at 72 hours after Aβ and sRAGE, pyridoxamine or ALT-711 were co-injected compared to those administered with Aβ alone (Figs. 5A, B). We also studied whether the activated microglia produces AGE-albumin in rat brains. Triple labeling confocal microscopy revealed that the relative numbers of AGE, albumin and Iba1 positive cells were increased in Aβ–injected rat brains, but decreased in Aβ/sRAGE exposed rat brains (Figs. 5C, D).

To investigate whether Aβ injection induces neuronal death and sRAGE protects RAGE-mediated neuronal death, triple labeling confocal microscopic analysis was performed. The relative numbers of RAGE positive neurons were increased dramatically after Aβ injection but decreased markedly after Aβ/sRAGE co-injection into rat brains (Figs. 5E, F). Consistently, the numbers of Bax- and p-JNK-positive neurons were increased markedly after Aβ injection but decreased in Aβ/sRAGE co-injected rat brains (Figs. 5G–I).

## Discussion

Many investigators reported importance of activated microglial cells in various neurodegenerative diseases [1], [2], [3]. However, it is poorly understood how activated microglia cells promote neuronal cell damage. We recently reported that albumin can be synthesized in microglial cells in the brain [6]. Mass spectral analysis further confirmed the synthesis of albumin. We also demonstrated that the synthesis and extracellular secretion of albumin from microglial cells were elevated upon microglial activation following Aβ1–42 exposure or lipopolysaccharide [6]. Since we did not know the physiological role of albumin in microglia cells, we initially proposed that albumin production would be protective against cell death by suppression of Aβ polymerization with enhancement of its clearance [6]. Contrary to our expectation, we now find a detrimental role of albumin synthesized in microglial cells. Our current results show that albumin alone does not affect cell death rate or Aβ polymerization/aggregation. In contrast, we demonstrate that albumin synthesized mainly from activated microglial cells is conjugated with AGE to produce a potently toxic AGE-albumin, which promotes Aβ polymerization and death of neuronal cells in primary culture. This conclusion was further supported by the results obtained from animal experiments as well as brain specimens from AD individuals compared to the corresponding controls.

To study the mechanism by which AGE-albumin synthesis is increased and how it promotes neuronal cell death, we first investigated the distribution of AGE and albumin in HMO6 microglial cells and human AD brains. Surprisingly, most AGEs were co-localized with albumin (Fig. 1), suggesting that AGE-albumin could be a major AGE product in microglial cells in the brain. Interestingly, the double-labeled AGE-albumin signal was highly localized in the vicinity of the cells with apoptotic nuclei. Based on our data of the elevated levels of AGE-albumin and co-localization with apoptotic cells, we concluded that AGE-albumin, the most abundant protein modified by AGE, is produced largely by microglial cells but not other cell types in the rat and human brains. Moreover, immunoblot analysis of whole cell lysates revealed that the rate of AGE-albumin synthesis in HMO6 microglial cells was markedly increased in a concentration-dependent manner following Aβ exposure. Taken together, these results strongly indicate that albumin is synthesized and secreted mostly as AGE-albumin from microglial cells, and that both the synthesis and secretion of AGE-albumin are significantly increased by Aβ treatment.

Aβ1–42, which increases AGE-albumin synthesis, is known to exert its toxicity through increased oxidative stress [16]. In our data, the amounts of both intracellular and secreted AGE-albumin, but not albumin itself, positively correlated with the degree of oxidative stress. Based on the data from hydrogen peroxide and ascorbic acid experiments, we conclude that Aβ–mediated increased oxidative stress is responsible for the synthesis and its extracellular secretion of AGE-albumin in human HMO6 microglial cells.

Several studies reported that AGEs are localized in the senile plaques and extra-cellular spaces [8], [9], [11], [13], [16], [25]. In addition, albumin was known as a potent inhibitor of Aβ polymerization and the amyloid inhibitory activity isolated from CSF and plasma was ascribed to the presence of albumin [25], suggesting that albumin may directly interact with Aβ. Our results show that AGE-albumin is closely associated with Aβ in HMO6 microglial cells, in neurons of Aβ-exposed rat brains, and human AD brains. But it is largely unknown whether the amount and distribution of AGE-albumin, the most abundant and modified protein synthesized in microglial cells, correlate positively with amyloid plaques. Our data indicate that AGE-albumin increases microglial Aβ synthesis and accumulation in a vicious cycle, which further aggravates the AD conditions through increased Aβ production via up-regulating the BACE level, AGE-albumin synthesis, and neuronal cell death. Furthermore, our data showed that the increased Aβ accumulation is likely promoted through inactivation of a chaperone PDI, as exemplified in the brains of AD individuals, leading to Aβ accumulation [15].

Increased level of RAGE, known to be expressed in neurons, highly correlated with neuronal death and development of AD [16]. In addition, AGE binds to RAGE in primary neurons [25]. Our immunohistochemical and immunoblot data showed that the amount of RAGE was significantly increased in primary neurons exposed to AGE-albumin compared to those untreated or treated with albumin alone. Since the stress-activated protein kinases and increased mitochondrial calcium influx are critically important in initiating apoptosis [26], [27], we also evaluated the effects of AGE-albumin on the respective changes of MAPKs, Bax, mitochondrial calcium influx, and cell death rate in the human primary neurons. Our data demonstrate that AGE-albumin directly promotes apoptosis of neuronal cells through activating the calcium-JNK-Bax pathway, as demonstrated previously in other cell types [28], [29].

The sRAGE is an extracellular component of RAGE and can inhibit AGE-RAGE interaction by binding AGE in extracellular spaces [11], [16]. Our results revealed that sRAGE protected Aβ–induced neuronal death when it was injected into rat brain together with Aβ. We also showed that the activated microglia produces AGE-albumin in rat brains after Aβ injection. Moreover, the number of AGE-albumin positive activated microglia was also decreased in Aβ/sRAGE co-injected rat brains. The relative numbers of neurons positive with RAGE, Bax, and p-JNK were increased dramatically in Aβ injected rat brains but markedly decreased after Aβ/sRAGE co-administration. These *in vivo* results were consistent with our in vitro data observed with human microglial cell line or human primary neuronal cells.

In summary, our current data show for the first time that AGE-albumin, the most abundant form of brain AGEs, is synthesized in microglial cells and secreted into extracellular space. The rate of AGE-albumin synthesis is markedly increased by Aβ treatment and increased oxidative stress while its elevated levels are frequently observed in the human brains of AD individuals compared with controls. AGE-albumin also promotes Aβ aggregation in microglial cells. Furthermore, AGE-albumin promotes the calcium-JNK-Bax-mediated apoptosis in primary neurons from AD individuals (Figure S2). Our results, therefore, provide a new mechanistic insight by which microglial cells play an important role in promoting neuronal death in human primary cells from AD individuals and Aβ-exposed rat brains by synthesizing and secreting potentially toxic AGE-albumin. Finally, AGE-albumin could be an excellent biomarker as a therapeutic target for neurodegenerative diseases including Alzheimer’s disease.

## Materials and Methods

### Cell Culture

For in vitro studies, an immortalized human microglial cell line (HMO6) was used. HMO6 cells were grown in Dulbecco’s modified Eagle’s medium (DMEM, Gibco) containing a high glucose concentration supplemented with 10% heat-inactivated fetal bovine serum (FBS, Gibco) and 20 mg/ml gentamycin (Sigma-Aldrich). These cells were maintained at 37°C under 5% CO_2_. HMO6 cells were exposed to Aβ1–42 (Sigma-Aldrich) at concentrations from 0 up to 400 or 5 nM. For inhibition studies, HMO6 cells were exposed to pyridoxamine (Sigma-Aldrich) for 3 h at concentrations from 0 up to 1 µM or ALT-711 (BioTrader) for 1 h at concentrations from 0 up to 5 µM before 5 nM Aβ1–42 treatment. HMO6 cells were then harvested 6 h after Aβ1–42 treatment for further analysis.

### Primary Culture of Human Neuronal Cells

Primary human neuronal cells were prepared from human brain tissues. The brain tissue collection and usage were approved by the Ethics Committee of the Seoul National University College of Medicine, Seoul, Korea. Human primary neurons were prepared as previously described [30]. In brief, small pieces of human brain cortexes were incubated with phosphate-buffered saline (PBS) containing 0.25% trypsin and 40 mg/ml DNase I for 30 min at 37°C. Dissociated cells were suspended in 5% decomplemented serum in high glucose-containing minimal essential medium with Earle’s salts, 1 mM sodium pyruvate, and 2 mM glutamine. All glial cells were removed and these neuronal cells were maintained at 37°C under 5% CO2 for further experiment.

### Human Neuronal Cells

Primary human neuronal cells were purchased from ScienceCell Research Laboratories (HN 1520). Human neuronal cells were grown in Neuronal Medium (NM 1521) according to the manufacturer’s suggestion for 2 days before AGE-albumin treatment.

### AGE-albumin and Aβ

AGE-albumin (A9810) and monomeric Aβ1–42 (A8301) were purchased from Sigma-Aldrich. Oligomeric Aβ was produced from monomeric Aβ by the previously published method [31], [32].

### Immunocytochemistry (ICC)

Cells were grown on Lab-Tek II slide chambers (Nunc), rinsed with PBS, fixed in methanol for 15 min, and rinsed again with PBS. The fixed cells on slide chambers were incubated overnight at 4°C with the following primary antibodies: rabbit anti-AGE antibody (1∶200, Abcam), mouse anti-human-albumin antibody (1∶200, R&D System), anti-BACE antibody (1∶50, Santa Cruz), anti-ADAM10 antibody (1∶200, R&D System), anti-APP antibody (1∶200, Chemicon), anti-RAGE antibody (1∶50, Santa Cruz), or anti-mitochondria antibody (1∶50, Abcam). After overnight incubation, the primary antibodies were washed with PBS three times and the slides were incubated for 1 h at room temperature with one of the following secondary antibodies: Alexa Fluor 633 anti-mouse IgG (1∶500, Invitrogen), Alexa Fluor 488 anti-rabbit IgG (1∶500, Invitrogen), or Alexa Fluor 555 anti-goat IgG (1∶500, Invitrogen). After washing the secondary antibodies with PBS three times at 10-min intervals, coverslips were mounted onto glass slides using the Vectashield mounting medium (Vector Laboratories), and examined under a laser confocal fluorescence microscope (LSM-710, Carl Zeiss).

### Animals

Thirty adult male Sprague-Dawley rats (230–350 g) were used in this study. The rats were maintained on a 12-h light-dark cycle, had access to food and water *ad libitum*, and were acclimated for at least 1 week prior to usage. All animal experiments were approved by the Institute Animal Care and Use Committee of Lee Gil Ya Cancer and Diabetes Institute of Gachon University and conducted humanely.

### Entorhinal Cortex Aβ or sRAGE (Soluble RAGE) or ALT-711 Injection

Animals were anaesthetized with ketamine HCl (0.75 mg/kg body weight) and xylazine (1 mg/kg body weight) prior to surgical procedures. For *in vivo* treatments, Aβ1–42 peptide was dissolved in the artificial cerebrospinal fluid (ASCF, from Tocris Bioscience) at a concentration of 400 µM and kept at 4°C until use. Aβ1–42 was injected into the entorhinal cortex (EC) with the aid of a stereotaxic instrument, following the midline incision of the scalp skin. The skull was pierced with a biological electric drill at the bregma (posteriorly, 8.3 mm; laterally, 5.4 mm) and the needle (30 gauge) on a 5 µL Hamilton syringe was lowered vertically until it reaches the target areas (depth, 4.5 mm). Three microliters of 200 µM Aβ1–42 diluted in ASCF or 3 µL of ASCF (as a negative vehicle control) were injected slowly at the rate of 1 µL per minute with an automatic microinjector. Then the syringe was removed slowly and surgical wounds were sutured with wound clips followed by topical treatment with antibiotics. To determine the protection by sRAGE or ALT-711, five rats were co-injected with Aβ1–42 and 3 µL of 6.7 nM sRAGE or 3 µL of 40 mM ALT-711.

### Tissue Preparation

Most rats were allowed to recover for a total of 3 days post injection. After full recovery, all rats were re-anaesthetized by the same manner and perfused trans-cardially with 100–200 mL of heparinized saline at 18°C followed by 400 mL of 4% paraformaldehyde-lysine periodate in 0.1 M sodium phosphate buffer (pH, 7.4). The brains were removed, placed in the same fixative for 4 h at 4°C, and then transferred into ice-cold 0.1 M phosphate-buffered saline (PBS) containing 20% sucrose. The brains were cut in a transverse plane at 10 µm thickness with a freezing microtome and were stored at −80°C until use.

### Immunohistochemistry (IHC)

Human brain tissues from normal adults and AD individuals were obtained from the Brain Bank of Seoul National University Hospital and the Brain Bank of Niigata University Hospital. The collection and use of human brain tissues were approved by the Institutional Review Board of Clinical Research Institute, Seoul National University Hospital and Niigata University Hospital, respectively. Brain tissues were fixed in 4% paraformaldehyde in 0.1 M neutral phosphate buffer, followed by cryoprotection in 30% sucrose solution overnight, and then 10 µm sections were prepared with a cryostat (Leica CM 1900). Paraffin-embedded brain tissues were cut into 4 µm-thick sections, deparaffinized with xylene, and rehydrated with a series of graded ethanol. Normal goat serum (10%) was used to block non-specific protein binding. Other staining methods were the same as described in the immunocytochemistry.

### Enzyme-linked Immunosorbent Assay (ELISA)

The amounts of AGE-albumin or Aβ in the extracellular culture media and cell lysates were determined by double ELISA using rabbit anti-Aβ, anti-AGE antibody and mouse anti-human ALB antibody. Six biological replicates were used, and each sample was measured in duplicate. We coated the 96-wells by incubation with an anti-ALB antibody (1∶800, Abcam). The unbound anti-ALB antibody was washed with 1×PBS three times at 10 min intervals. Filtered extracts using Amicon filter (cut-off 3 kD) from culture media or cell lysates were then added into each well and incubated for 1 h to be captured by the bound anti-ALB antibody. The unbound proteins were washed off from each well by washing with 1×PBS three times at 10 min intervals. After washing the unbound ALB, the second anti-AGE antibody (1∶1000, Abcam) was added to each well to allow interaction with AGE of the captured ALB-AGE. The unbound anti-AGE antibody was then washed off with 1×PBS three times before HRP conjugated anti-rabbit secondary antibody (1∶1000, Vector Laboratories) was incubated for additional 1 h. After washing the unbound HRP-conjugated secondary antibody, color was developed by incubation with 3,3′, 5,5′-tetramethylbenzidine (TMB) for 15 mins, and reaction stopped with an equal volume of 2 M H_2_SO_4_. Absorbance in each well was measured at 450 nm using an ELISA plate reader (VERSA Max, Molecular Devices).

### Co-immunoprecipitation

Whole cell lysates from HMO6 before and after Aβ treatment were prepared with RIPA buffer containing 1 M Tris (pH 7.5), 5 M NaCl, 10% NP-40, 10% deoxycholate and protease inhibitor cocktail (Calbiochem). The experiment was repeated three times. Whole cell lysates (0.5 mg protein) were incubated with 5 µg of anti-AGE (Abcam) or anti-protein disulfide isomerase (PDI) for 4 h followed by overnight incubation with protein G-agarose in 500 µl PBS overnight at 4°C. The agarose beads were precipitated by centrifugation at 14,000 rpm for 5 min, and washed three times with 1 ml washing buffer containing 50 mM Tris–Cl and 500 mM NaCl (pH 8.0). The immunoprecipitated proteins were resolved on a 4–12% polyacrylamide gel (Invitrogen), and subjected to immunoblot analysis with the respective antibody as follows: anti-AGE antibody (1∶1000, Abcam), anti-albumin antibody (1∶1000, Abcam), anti-PDI antibody (1∶200, Santa Cruz), or anti-*S*-NO-Cys antibody (1∶200, Sigma).

### Immunoblot Analysis

Whole cell lysates were prepared with RIPA buffer containing 4% CHAPS. Proteins from each group were separated in 4–12% polyacrylamide gels (Life Technology) and transferred to nitrocellulose membranes. The primary antibodies used were: anti-BACE (1∶200, Santa Cruz); anti-ADAM10 (1∶1,000, R&D System); anti-APP (1∶1,000, Chemicon); anti-RAGE (1∶200, Santa Cruz), and anti ß-actin (1∶1,000, Cell Signaling).

### ThT Fluorescence Assay

ThT fluorescence assays were performed with synthetic Aβ1–42 (Sigma-Aldrich) as described previously with constant shaking for 2.5 h at 37°C. Albumin and AGE-albumin were added at 10 µM each. ThT emission fluorescence was measured at 483 nm (450 nm excitation) with a Perkin-Elmer luminescence spectrophotometer LS-55. Fluorescence values for albumin- or AGE-albumin-exposed cells were normalized to the DMSO-treated negative control and expressed as percentage relative fluorescence.

### Apoptosis Detection Assay

HT Titer TACS Assay kit (R&D systems) was used to detect apoptotic neuronal cells after AGE-ALB treatment by following the manufacturer’s protocol. Briefly, human neuronal cells grown in 96-well plates were fixed with 4% formaldehyde for 7 min followed by proteinase K incubation for 15 min. The neuronal cells in each well were then labeled with TdT (Terminal deoxynucleotydil transferase) for 60 min and washed to remove the excess TdT reagent. The cells were incubated with Streptavidin-HRP for 1 h, followed by removal of the unbound Streptavidin-HRP, the HRP enzyme reaction was performed by TACS-Sapphire. By stopping the reaction with 5% phosphoric acid, apoptotic cells in each plate were determined by fluoro-cytometry at 450 nm.

### Calcium Imaging

Primary human neuronal cells were grown on Lab-Tek II glass slide chambers (Nunc). After 2 days of cell culture, cells were incubated with 4 µM Fluo-3 dye (Life Technology) for 40 min at 37°C. After incubation with Fluo-3 dye, the cells were subjected to image analysis with a laser confocal fluorescence microscope (LSM 710, Carl Zeiss). Upon adjusting proper fields, 100 ng/ml of AGE-albumin was carefully added into the slide chamber to record any changes in intracellular calcium levels during the first 20 min.

### Densitometry and Statistical Analysis

The densitometric intensity of each immunoreactive band was determined by using a gel digitizing Image-Pro software. All data in this report represent the results from at least 3 independent experiments, unless stated otherwise. Statistical analyses were performed using the Student’s *t* test and *p*<0.05 was considered statistically significant.

## Supporting Information

Figure S1 Increased Aβ synthesis through up-regulation of BACE following AGE-ALB treatment. (A) The relative amounts of APP, ADAM10, or BACE were studied by immunoconfocal microscopic image analysis after HMO6 cells were treated with or without AGE-albumin (AGE-ALB). Scale bar = 50 µm. (B, C) Whole cell lysates (0.01 mg protein/lane) were used to determine the levels of BACE, ADAM10, and APP in HMO6 cells before or after AGE-ALB treatment by immunoblot analysis. β-Actin level is shown for comparable protein loading per lane. (D, E) Whole cell lysates (0.01 mg protein/lane) of HMO6 cells after AGE-albumin exposure were prepared and used for immunoblot analysis to determine the levels of Aβ.
(PDF)

Figure S2 A proposed model of AGE-albumin mediated neuronal cell death and its contribution to AD. The schematic diagram illustrates the synthesis in microglial cells and extracellular secretion of AGE-albumin, which induces neuronal cell death and ultimately contributes to neurodegeneration. AGE-albumin synthesis and secretion in microglial cells is increased upon Aβ treatment. Consequently, elevated amounts of AGE-albumin are ubiquitously distributed in the brain cortex of AD individual. AGE-albumin then increases RAGE expression and mitochondrial calcium influx, leading to apoptosis in primary neurons. The microglial cells may also play an important role in neuronal cell death in AD by synthesizing and secreting AGE-albumin, which promotes Aβ production and aggregation in microglial cells. Taken together, AGE-albumin promotes death of primary neuronal cells, and of neurons in rat brains, and human brains, likely contributing to neurodegenerative diseases including AD.
(PDF)
