# Supplementary figures and images for: Induction of Neuronal Death by Microglial AGE-Albumin: Implications for Alzheimer’s Disease
Source: PLoS One. 2012 May 25;7(5):e37917. doi: 10.1371/journal.pone.0037917 (PMC3360664; doi:10.1371/journal.pone.0037917)

Figure S1

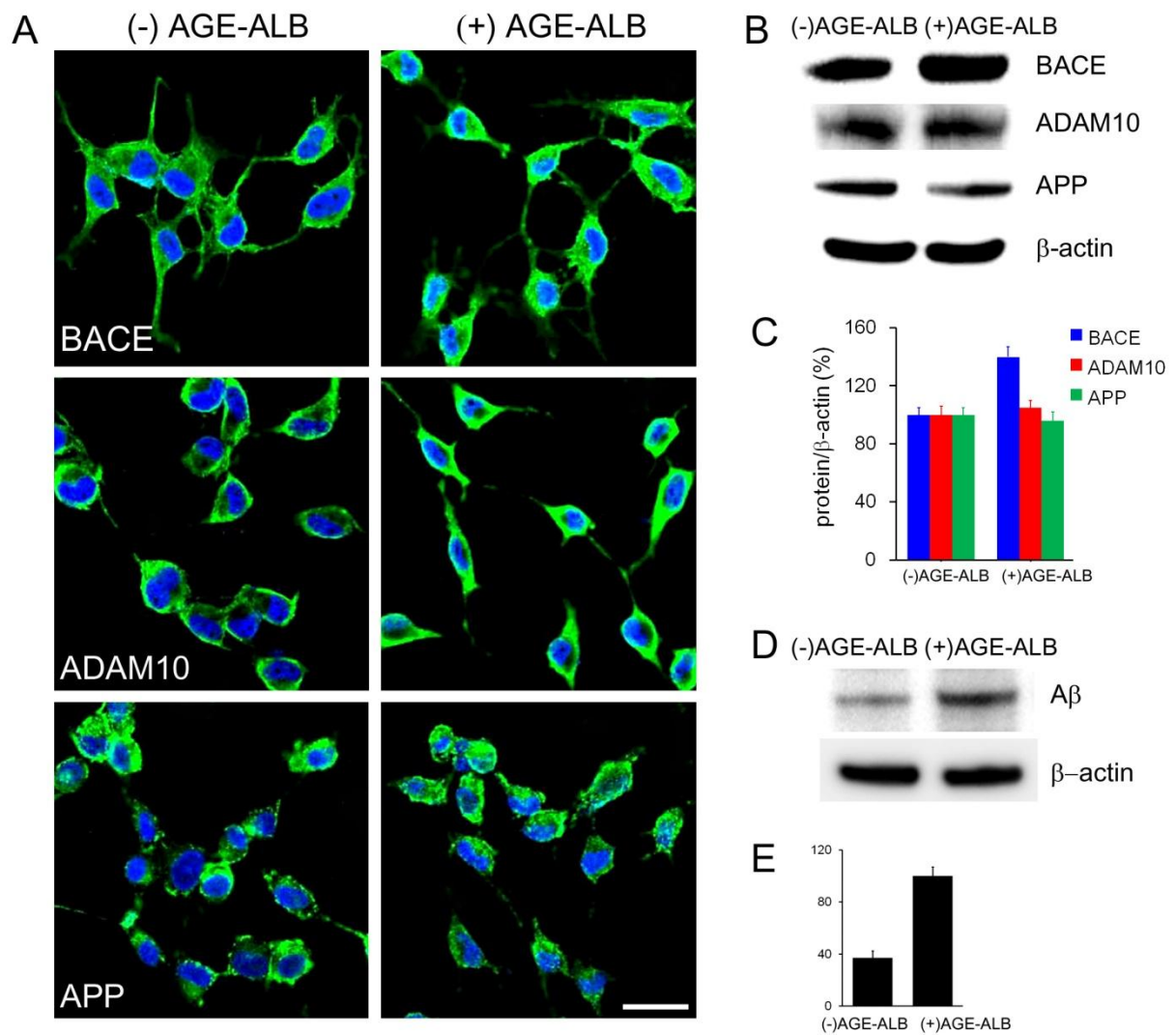

Supplement: Figure S1 — Increased Aβ synthesis through up-regulation of BACE following AGE-ALB treatment. (A) The relative amounts of APP, ADAM10, or BACE were studied by immunoconfocal microscopic image analysis after HMO6 cells were treated with or without AGE-albumin (AGE-ALB). Scale bar = 50 µm. (B, C) Whole cell lysates (0.01 mg protein/lane) were used to determine the levels of BACE, ADAM10, and APP in HMO6 cells before or after AGE-ALB treatment by immunoblot analysis. β-Actin level is shown for comparable protein loading per lane. (D, E) Whole cell lysates (0.01 mg protein/lane) of HMO6 cells after AGE-albumin exposure were prepared and used for immunoblot analysis to determine the levels of Aβ. (PDF) [file pone.0037917.s001.pdf]

Figure S2

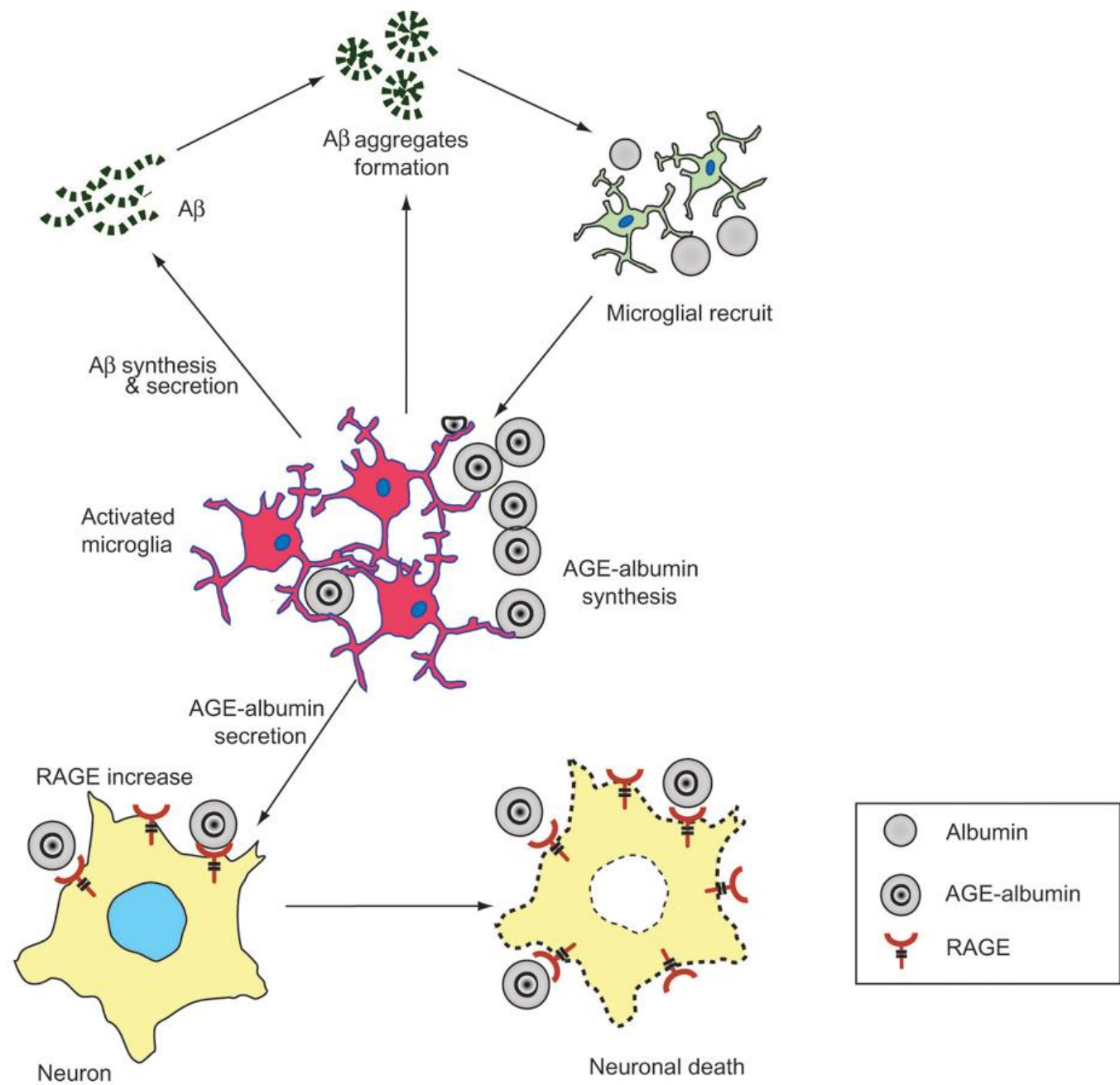

Supplement: Figure S2 — A proposed model of AGE-albumin mediated neuronal cell death and its contribution to AD. The schematic diagram illustrates the synthesis in microglial cells and extracellular secretion of AGE-albumin, which induces neuronal cell death and ultimately contributes to neurodegeneration. AGE-albumin synthesis and secretion in microglial cells is increased upon Aβ treatment. Consequently, elevated amounts of AGE-albumin are ubiquitously distributed in the brain cortex of AD individual. AGE-albumin then increases RAGE expression and mitochondrial calcium influx, leading to apoptosis in primary neurons. The microglial cells may also play an important role in neuronal cell death in AD by synthesizing and secreting AGE-albumin, which promotes Aβ production and aggregation in microglial cells. Taken together, AGE-albumin promotes death of primary neuronal cells, and of neurons in rat brains, and human brains, likely contributing to neurodegenerative diseases including AD. (PDF) [file pone.0037917.s002.pdf]
